# Predictive Value of Sustained Virologic Response at Week 4 in Patients with Hepatitis C Virus Infection Treated with Sofosbuvir/Velpatasvir

**DOI:** 10.3390/v18020269

**Published:** 2026-02-21

**Authors:** Gia Landry, Mark Sulkowski, Jordan J. Feld, Nancy Reau, Stacey Scherbakovsky, Farrah Black, Candido Hernández, Renee-Claude Mercier, Liyun Ni, Marc Bourlière, Alessandra Mangia

**Affiliations:** 1 Lane Regional Medical Center, Zachary, LA 70791, USA; 2 Louisiana State University Health, Baton Rouge, LA 70805, USA; 3 Department of Medicine, Johns Hopkins University School of Medicine, Baltimore, MD 21287, USA; 4 Toronto Centre for Liver Disease, University Health Network, University of Toronto, Toronto, ON M5G 2C4, Canada; 5 Rush University Medical Center, Chicago, IL 60612, USA; 6 Gilead Sciences, Inc., Foster City, CA 94404, USArenee-claude.mercier@gilead.com (R.-C.M.);; 7 Hôpital Saint Joseph, 13008 Marseille, France; 8 INSERM UMR 1252 IRD SESSTIM Aix Marseille Université, 13385 Marseille, France; 9 Liver Unit, IRCCS Fondazione “Casa Sollievo della Sofferenza”, 71013 San Giovanni Rotondo, Italy

**Keywords:** chronic hepatitis C, concordance, direct-acting antiviral agents, hepatitis C, loss to follow-up, marginalized populations, sustained virologic response, sustained virologic response at 4 weeks post-treatment

## Abstract

Direct-acting antiviral therapies can cure most people with hepatitis C virus (HCV) infection with little need for testing or monitoring. A major challenge to eliminating HCV is ensuring patients complete all steps of care, including confirmation of cure. We assessed the concordance of sustained virologic response (SVR) at 4 weeks (SVR4) and 12 weeks (SVR12) post-treatment to evaluate the viability of SVR4 as a predictor of cure in patients treated with sofosbuvir (SOF)/velpatasvir (VEL). We conducted a retrospective analysis of patients from the Phase 3 ASTRAL-1, -2, and -3 programs and a historical cohort from the Louisiana Department of Health Sexually Transmitted Infection (STI)/HIV/Hepatitis Program claims database. Concordance analyses were performed for patients with both SVR4 and SVR12 data. The concordance analysis in the ASTRAL studies included 1015 patients; 1005 and 1002 achieved SVR4 and SVR12, respectively. Among SVR4 achievers, 3 failed to maintain SVR12, while all (10/10) patients who did not achieve SVR4 also failed SVR12. In the real-world cohort, 479/509 (94%) patients achieved SVR4 and 485/509 (95%) achieved SVR12. Of those with SVR4, 7 failed SVR12; 17 of 30 patients who did not achieve SVR4 also failed SVR12. High concordance between SVR4 and SVR12 was observed in both ASTRAL and the real-world dataset, supporting the use of SVR4 as a predictor of long-term SVR in patients with HCV infection treated with SOF/VEL. Streamlining cure confirmation by shifting SVR determination from week 12 to week 4 post-treatment may reduce patient loss to follow-up.

## 1. Introduction

Chronic hepatitis C virus (HCV) infection can lead to cirrhosis, decompensated liver disease, and hepatocellular carcinoma [1]. According to the World Health Organization (WHO), approximately 50 million people worldwide have chronic HCV infection, with an estimated 1 million individuals newly infected each year [2]. Over the past decade, direct-acting antiviral (DAA) therapies have revolutionized HCV treatment, offering a combination of high efficacy, safety, tolerability, and simplicity [1,3]. The WHO set a target to eliminate HCV as a public health threat by 2030, and the availability of DAA therapies has made this goal achievable [4].

International guidelines for HCV treatment recommend pangenotypic DAA regimens, such as glecaprevir/pibrentasvir or sofosbuvir (SOF)/velpatasvir (VEL) [5,6,7]. These regimens have enabled most patients who are diagnosed and access treatment to achieve a cure with minimal pretreatment testing and on-treatment monitoring [7,8,9]. The WHO, the American Association for the Study of Liver Diseases and the Infectious Diseases Society of America (AASLD-IDSA), and the European Association for the Study of the Liver (EASL) have endorsed the simplification of treatment as part of a global call to action for HCV elimination [5,6,7,10].

While DAA regimens and simplified guidelines have streamlined HCV testing and treatment, ensuring effective follow-up and confirmation of cure remains crucial, particularly for individuals with high-risk behaviors or those susceptible to reinfection [5,7]. Although EASL guidelines suggest that sustained virologic response (SVR) testing can be omitted in some cases [5], it also provides an opportunity to counsel patients on the risk of reinfection and the importance of regular testing for those with ongoing risk factors [4,11]. Patient loss to follow-up (LTFU), especially during treatment, is a major barrier in the care cascade; LTFU rates among patients with HCV are commonly reported to be near 10%, although rates as high as 65% have been observed [12,13,14,15]. Risk factors for LTFU include male sex, younger age, drug or alcohol use disorder, psychiatric illness, and housing insecurity [12,16,17,18].

Simplifying HCV treatment is particularly important for marginalized populations, who often face significant barriers to accessing care [6,19]. Groups such as people who inject drugs, those experiencing homelessness, and those with a history of incarceration are disproportionately affected by HCV [20,21,22]. An estimated 52% to 67% of people who inject drugs are infected with HCV [4,20,21]. In prisons worldwide, the prevalence of HCV infection can exceed 10%, with some countries having rates as high as 32% [23,24].

HCV cure was historically defined as achieving SVR with undetectable HCV RNA at 24 weeks after treatment completion (SVR24) [25]. However, studies demonstrating a strong concordance between SVR at 12 weeks (SVR12) and SVR24, particularly with treatments such as interferon monotherapy and pegylated interferon plus ribavirin [26,27], have shown that virologic relapse beyond SVR12 is rare, leading to the adoption of SVR12 as a standard endpoint for cure [5]. EASL and AASLD-IDSA guidelines now define HCV cure based on either SVR12 or SVR24, while the Food and Drug Administration uses SVR12 [5,7,28].

Strong concordance between SVR at 4 weeks (SVR4) and SVR12 has been shown for several DAA regimens, including glecaprevir/pibrentasvir [29,30,31], ledipasvir/SOF [30,31], and SOF/VEL [30,31,32]. Australia and Saudi Arabia have introduced guidelines recommending the use of SVR4, particularly for patients at risk of LTFU [33,34]. The International Network on Health and Hepatitis in Substance Users (INHSU)—Prisons has implemented global guidelines recommending the use of SVR4 in prison settings [35]. Additionally, AASLD-IDSA guidance acknowledges SVR4 as an acceptable alternative time point to assess HCV cure for people without cirrhosis or prior DAA exposure [36,37].

A recent study of global HCV treatment patterns estimated that approximately 12.7 million people across 119 countries received DAA therapy between 2014 and 2023 [38]. Of these, roughly 11.1 million (87%) were treated with a SOF-based regimen. Given the global burden of chronic HCV, estimated at 50 million people [2], this suggests that approximately three-quarters of those affected have yet to receive DAA treatment. Furthermore, 67% of DAA treatments during this period were concentrated in just 4 regions: Egypt, Pakistan, the United States, and the European Union [38]. These findings indicate there is an ongoing need to improve DAA accessibility and simplify HCV treatment worldwide. In this study, we evaluated the concordance of SVR4 with SVR12 in patients receiving SOF/VEL in the ASTRAL-1, -2, and -3 Phase 3 randomized clinical trials (RCTs) as well as in a real-world setting by utilizing Medicaid claims from the Louisiana Department of Health Sexually Transmitted Infection (STI)/HIV/Hepatitis Program administrative claims database. Our objective was to assess the predictive value of SVR4 for HCV cure for patients treated with SOF/VEL in an RCT and a real-world setting.

## 2. Materials and Methods

### 2.1. Randomized Clinical Trials

**Figure 1:**
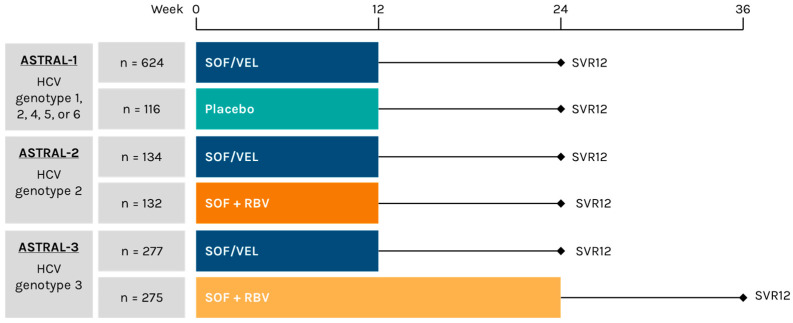
Sofosbuvir (SOF)/velpatasvir (VEL) in the ASTRAL-1, -2, and -3 Phase 3 programs. HCV, hepatitis C virus; RBV, ribavirin; SVR12, sustained virologic response at 12 weeks post-treatment.

HCV RNA data from patients enrolled in ASTRAL-1 (NCT02201940), ASTRAL-2 (NCT02220998), and ASTRAL-3 (NCT02201953) were evaluated. Patients in ASTRAL-1 were enrolled at 81 sites in the United States, Canada, Europe, and Hong Kong [39]. ASTRAL-2 enrolled patients at 51 sites in the United States, and ASTRAL-3 enrolled patients at 76 sites in the United States, Canada, Europe, Australia, and New Zealand [40]. The study designs are shown in Figure 1; detailed methodologies for each study were published previously [39,40]. Participants in ASTRAL-4, which enrolled patients with decompensated cirrhosis [41], and ASTRAL-5, which enrolled patients with HCV and HIV-1 coinfection [42], were excluded. SVR was defined as an HCV RNA level below the lower limit of quantitation (15 IU/mL) 12 weeks after treatment, as measured by the COBAS AmpliPrep/COBAS TaqMan HCV Quantitative Test, version 2.0 (Roche Molecular Diagnostics, Pleasanton, CA, USA).

For the ASTRAL-1, -2, and -3 studies, eligible patients were aged 18 years or older and had chronic HCV infection with HCV genotype (GT) 1, 2, 3, 4, 5, or 6 [39,40]. Patients could be either treatment-naïve or treatment-experienced, with treatment-experienced patients defined as those who had previously failed a regimen containing interferon, with or without ribavirin, and completed treatment at least 8 weeks prior to baseline (day 1). The ASTRAL-1, -2, and -3 protocols aimed to enroll approximately 20% treatment-experienced patients and allowed for approximately 20% of participants to have compensated cirrhosis.

Exclusion criteria for the ASTRAL-1, -2, and -3 RCTs included current or historical evidence of hepatic decompensation or a diagnosis of hepatocellular carcinoma. Patients who had discontinued any prior HCV treatment due to an adverse event or who had previously been treated with any nucleotide analog HCV nonstructural protein 5B (NS5B) inhibitor or any NS5A inhibitor were not eligible for enrollment. For this analysis, all patients enrolled in ASTRAL-1, -2, or -3 were included, although concordance analyses only included those with both SVR4 and SVR12 data.

All trials were conducted in accordance with the International Conference on Harmonisation Good Clinical Practice guidelines, the Declaration of Helsinki, and local regulations. The protocols were approved by the relevant institutional review boards or independent ethics committees.

### 2.2. Real-World Data

A historical cohort was analyzed using comprehensive medical and pharmacy Medicaid claims from the Louisiana Department of Health STI/HIV/Hepatitis Program administrative claims database, covering the period from 15 July 2019 to 31 December 2024. This hepatitis surveillance program collects detailed information, including patient demographics (e.g., sex, gender, age, ethnicity, and race), diagnostic testing information (e.g., results of HCV antibody tests, qualitative and quantitative HCV RNA tests, and HCV GT), and clinical data (e.g., comorbid conditions, pregnancy status, medications taken, dates of use, and duration). Only treatment-naïve patients were included in the cohort.

The cohort for this analysis included adults aged 18 years or older enrolled in Medicaid who had at least 1 HCV diagnosis (chronic or acute) and who initiated SOF/VEL treatment during the study period. Patients with cirrhosis were not excluded. Measurements of SVR4 were taken between 4 and 6 weeks post-treatment, while measurements of SVR12 were taken at least 10 weeks post-treatment. For the concordance analysis, only patients with both SVR4 and SVR12 data were included.

As the data obtained from the Louisiana Department of Health are de-identified and publicly available, this analysis does not involve human subjects research and therefore did not require approval by an institutional review board.

### 2.3. Statistical Analysis

Concordance analyses included only patients treated with SOF/VEL who had both SVR4 and SVR12 data, with no imputation of missing data. Positive predictive value (PPV) was defined as the proportion of patients achieving SVR12 among those with SVR4. Negative predictive value (NPV) was defined as the proportion of patients not achieving SVR12 among those who did not achieve SVR4. Sensitivity was calculated as the proportion of patients achieving SVR4 among those achieving SVR12. Specificity was calculated as the proportion of patients not achieving SVR4 among those not achieving SVR12. All statistical analyses were performed using SAS software version 9.4.

## 3. Results

### 3.1. Randomized Clinical Trial Data from the ASTRAL Trials

A total of 1035 patients received SOF/VEL in the ASTRAL-1, -2, and -3 studies. The majority were male, and the mean age was 53 years; 28% were treatment-experienced, and 21% had cirrhosis (Table 1). All HCV GTs were represented, with the most common being GT1 (32%), GT2 (23%), and GT3 (27%).

Among the 1035 patients who received SOF/VEL, 20 did not achieve SVR12. Of these, 12 patients experienced virologic relapse, 1 was reinfected, 4 were LTFU, 1 discontinued SOF/VEL treatment, 1 withdrew consent, and 1 died, with the death considered unrelated to treatment. Among the 13 patients who relapsed or were reinfected, 10 experienced these events by post-treatment week 4, and 3 by week 12 (Table 2). Of the 10 patients who relapsed or were reinfected before post-treatment week 4, 8 had HCV GT3 and 1 each had HCV GT1a and GT1b. All patients who relapsed or were reinfected between post-treatment weeks 4 and 12 had HCV GT3a. Additionally, 8 of these 13 patients had compensated cirrhosis, and 8 had prior treatment with pegylated interferon plus ribavirin.

**Figure 2:**
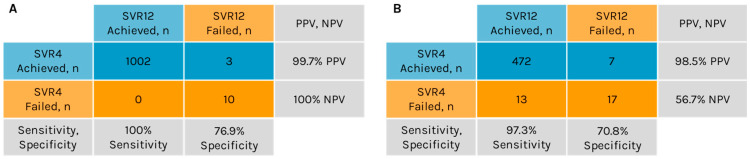
Concordance of sustained virologic response (SVR) at 4 weeks post-treatment (SVR4) and at 12 weeks post-treatment (SVR12) in the ASTRAL studies (**A**) and in the Real-World Hepatitis Surveillance Program (**B**). NPV, negative predictive value; PPV, positive predictive value.

A concordance analysis for SVR4 and SVR12 was conducted for 1015 patients with available data. Of the 1005 patients who achieved SVR4, 1002 also achieved SVR12, yielding a PPV of 99.7% for SVR4 in predicting SVR12 (Figure 2A). Among the 10 patients who did not achieve SVR4, none attained SVR12, resulting in an NPV of 100% for SVR4 in predicting SVR12. Sensitivity, defined as the proportion of true positive outcomes, was 100% (1002/1002), while specificity, defined as the proportion of true negative outcomes, was 76.9% (10/13).

### 3.2. Real-World Data from the Louisiana Department of Health STI/HIV/Hepatitis Program

#### 3.2.1. Patient Demographics

A real-world analysis of 11,299 patients with HCV who were treated with SOF/VEL was conducted using data collected by the Louisiana Department of Health STI/HIV/Hepatitis Program. The majority of patients were male (60%), and the mean age was 47 years (Table 1). All patients were treatment-naïve. GT data were available for 10,704 (95%) patients; all HCV GTs were represented except GT5, with GT1 being the most prevalent (78%). Of the 11,299 patients, 702 (6%) and 8005 (71%) had SVR4 and SVR12 data available, respectively, while 509 (5%) had both (Table 3). Among the 702 patients with SVR4 data, 659 (94%) achieved SVR4, while 43 (6%) had virologic relapse or reinfection at week 4. For the 8005 patients with SVR12 data, 7405 (93%) achieved SVR12, and 600 (7%) had virologic relapse or reinfection at week 12.

#### 3.2.2. Concordance of SVR4 and SVR12

Data for both SVR4 and SVR12 were available for 509 patients, who were included in the concordance evaluation. The median (range) timing of SVR4 and SVR12 measurements for these patients was 5 (4–6) and 22 (10–256) weeks post-treatment, respectively. The majority were male, and the mean age was 47 years (Table 1). As in the overall cohort, all HCV GTs except for GT5 were represented in the concordance analysis population, with GT1 being the most common (59%). Overall, patient demographics and disease characteristics among the 509 patients with both SVR4 and SVR12 data available were similar to those of the overall population of patients with HCV.

Among the 509 patients, 479 (94%) achieved SVR4 (Table 3). Of those who achieved SVR4, 472 also achieved SVR12, resulting in a PPV of 98.5% for SVR4 as a predictor of SVR12 (Figure 2B). Of the 30 patients who did not achieve SVR4, 17 also did not attain SVR12, while the remaining 13 patients did achieve SVR12, resulting in an NPV of 56.7% for SVR4 as a predictor of SVR12. The sensitivity and specificity of SVR4 for predicting SVR12 were 97.3% (472/485) and 70.8% (17/24), respectively.

Of the 509 patients analyzed, 30 (6%) did not achieve SVR4, and 24 (5%) did not achieve SVR12. Thirteen patients did not achieve SVR4 but subsequently attained SVR12; of these, 12 (92%) were male, 11 (85%) were White, and 2 (15%) had cirrhosis (Table 4). Among the 24 patients who did not achieve SVR12, 7 achieved SVR4 and subsequently failed to achieve SVR12, and 17 did not achieve SVR4 or SVR12. Of these 24 patients, 19 (79%) were male, 11 (46%) were White, 9 (38%) were Black, and 5 (21%) had cirrhosis.

Of the 7 patients who achieved SVR4 but not SVR12, the median (range) timing to SVR12 testing was 43 (13–101) weeks. This was an almost 2-fold increase compared with the overall cohort with both SVR4 and SVR12 testing, in which the median (range) time to SVR12 testing was 22 (10–256) weeks. There were also increased percentages of people with substance use disorder (43%) and opioid use disorder (71%) among those who achieved SVR4 but not SVR12 compared with the overall cohort with SVR4 and SVR12 testing, in which 207 (41%) and 197 (39%) patients had substance and opioid use disorders, respectively (Table 1 and Table 4).

## 4. Discussion

Treatment with SOF/VEL has been shown to be highly effective in both clinical trials and real-world cohorts, benefiting diverse populations and positively impacting patient-reported outcomes [39,40,43,44,45,46,47,48,49,50]. Furthermore, SOF/VEL is well tolerated, with an extensive body of evidence supporting its safety in the treatment of chronic HCV infection [43,44,45,46,49].

We observed high concordance between SVR4 and SVR12 among patients treated with SOF/VEL in the ASTRAL program. Only 3 (0.3%) of the 1005 patients achieving SVR4 subsequently failed to achieve SVR12. All 3 of these patients had HCV GT3a; 2 had previously been treated with pegylated interferon plus ribavirin, and 2 had compensated cirrhosis. At 12 weeks post-treatment, each of these patients had HCV RNA levels greater than 5.1 log_10_ IU/mL (Table 2). In total, 12 patients who received SOF/VEL in the ASTRAL RCTs experienced virologic relapse. Additionally, 1 patient with HCV GT3a had a confirmed GT1a reinfection between post-treatment weeks 4 and 12.

Similar results were observed in the real-world data, which showed high concordance (98.5% PPV) between SVR4 and SVR12 in patients treated with SOF/VEL. Of 479 patients who achieved SVR4, 7 did not achieve SVR12. Among these 7 patients, 5 (71%) had opioid use disorder, 3 (43%) had substance use disorder, and 2 (29%) were coinfected with HIV. While the rate of HCV recurrence is low in most patients who achieve SVR [51], high-risk groups, including men who have sex with men, people coinfected with HIV, and people who inject drugs, have been shown to have higher rates of recurrence, most often due to reinfection [52,53,54]. Given that most of the 7 patients who achieved SVR4 but not SVR12 engaged in high-risk behaviors, these patients may have experienced reinfection rather than relapse. This possibility is further supported by the observation that the median time to SVR12 testing in this group was almost 2-fold longer than among all patients with both SVR4 and SVR12 testing, allowing more time for reinfection to occur. In people with ongoing high-risk behaviors, it is important to test for reinfection at least annually, regardless of whether SVR4 or SVR12 is used to document cure [11].

The PPV of SVR4 for predicting SVR12 was high for both the ASTRAL Phase 3 RCTs and the real-world program. However, the NPV was substantially lower in the real-world data (56.7%) than in the RCTs (100%). Of the 30 patients in the real-world data who did not achieve SVR4, 13 went on to achieve SVR12. It has been shown that some patients who relapse after DAA therapy will subsequently spontaneously clear HCV without additional therapy [55]. In the current study, post-treatment HCV RNA levels were unavailable for the patients who achieved SVR12, but not SVR4; however, these patients may have had very low HCV RNA levels at post-treatment week 4 and experienced spontaneous clearance by week 12. This highlights the importance of confirming SVR before initiating retreatment in patients who do not achieve SVR4, as some may still clear the virus by SVR12. The specificity of SVR4 for predicting SVR12 was also lower in the real-world data (70.8%) than in the RCTs (76.9%), but the sensitivity of SVR4 for predicting SVR12 was similar in both the RCTs and real-world data.

The high concordance observed between SVR4 and SVR12, both in Phase 3 RCTs and in the real-world dataset, supports the use of SVR4 as an indicator of HCV cure in patients treated with SOF/VEL. Adoption of SVR4 as an endpoint in HCV care would be consistent with the global efforts to simplify HCV treatment endorsed by the WHO, AASLD-IDSA, and EASL [5,6,7,10]. In addition to minimizing the risk of patients being LTFU, use of SVR4 instead of SVR12 could reduce the number of follow-up appointments required for patients who received SOF/VEL treatment, potentially lowering the cost of care for patients. Importantly, such a change would necessitate that healthcare providers counsel patients on the risk of HCV reinfection and the importance of regular testing for those with ongoing risk factors at the 4-week follow-up appointment.

Our study has several limitations. First, the ASTRAL-1, -2, and -3 Phase 3 RCTs excluded patients with active drug use within the past 12 months, though those on stable opioid substitution therapy were eligible. Additionally, the distribution of HCV GTs differed between the ASTRAL studies and the real-world dataset, and sample sizes for GT4, GT5, and GT6 were small, which may limit the generalizability of our findings for these subgroups. A major limitation of the real-world dataset is that, while 71% (8005/11,299) of patients had SVR12 testing data, only 5% (509/11,299) of patients had both SVR4 and SVR12 data available. Also, because this dataset is based on Medicaid claims, it has inherent constraints, including potential underestimation of comorbidities, such as cirrhosis, and a limited ability to distinguish between active and past use or to assess disorder severity for people with a diagnosis of opioid or substance use disorder. The lack of HCV RNA sequencing data also prevents differentiation between virologic relapse and reinfection in those who did not achieve SVR, which could affect our concordance analysis. However, the number of people who achieved SVR4 but not SVR12 was very small. Additionally, the limited availability of HCV RNA data precluded assessment of whether individuals who achieved SVR12 but not SVR4 may have experienced low-level viremia. Finally, the real-world cohort was composed entirely of Medicaid beneficiaries, which may limit generalizability. Nonetheless, there is no reason to expect substantially different results among Medicare or commercially insured populations. Across both the ASTRAL-1, -2, and -3 studies and the real-world dataset, most patients were treatment-naïve and did not have cirrhosis or HIV coinfection; as such, further studies of the concordance of SVR4 and SVR12 among these populations are warranted.

This study represents the first validation of the concordance between SVR4 and SVR12 in patients receiving SOF/VEL, comparing data from RCTs with real-world data. These results confirm that SVR4 reliably predicts long-term SVR and provide support for using SVR4 as the endpoint in post-treatment monitoring. Adoption of SVR4 as the standard endpoint for patients receiving SOF/VEL can streamline follow-up care and potentially help reduce the risk of patients being LTFU, particularly among vulnerable populations.

## Figures and Tables

**Table 1:** Demographics of Patients in the Phase 3 ASTRAL Studies and in the Real-World Hepatitis Surveillance Program.

	Phase 3 ASTRAL Studies		Real-World Hepatitis Surveillance Program	
	Total	SOF/VEL	All Patients with HCV Treated with SOF/VEL	Patients with SVR4 and SVR12
	N = 1558	n = 1035	N = 11,299	n = 509
Age, years, mean (SD)	53 (10.7)	53 (11.0)	47 (11.6)	47 (11.6)
Sex, n (%)				
Male	944 (61)	630 (61)	6761 (60)	312 (61)
Female	614 (39)	405 (39)	4538 (40)	197 (39)
Race, n (%)				
Asian	131 (8)	86 (8)	39 (<1)	1 (<1)
Black	85 (5)	61 (6)	3485 (31)	174 (34)
Hispanic ^a^	—	—	998 (9)	45 (9)
White	1307 (84)	867 (84)	6637 (59)	281 (55)
Other or not disclosed	35 (2)	21 (2)	140 (1)	8 (2)
Hispanic, ^a^ n (%)	107 (7)	68 (7)	—	—
BMI, kg/m^2^, mean (SD)	26.9 (5.30)	26.8 (5.07)	—	—
HCV GT, ^b^ n (%)				
GT1	393 (25)	328 (32)	8757 (78)	302 (59)
GT2	391 (25)	238 (23)	772 (7)	12 (2)
GT3	552 (35)	277 (27)	1099 (10)	25 (5)
GT4	138 (9)	116 (11)	62 (1)	1 (<1)
GT5	35 (2)	35 (3)	0 (0)	0 (0)
GT6	49 (3)	41 (4)	14 (<1)	1 (<1)
Baseline HCV RNA, log_10_ IU/mL, mean (SD)	6.3 (0.70)	6.3 (0.70)	—	—
Cirrhosis, n (%)	343 (22)	220 (21)	1490 (13)	87 (17)
OST at enrollment, n (%)	—	51 (5)	—	—
Opioid use disorder, ^c^ n (%)	—	—	4682 (41)	197 (39)
Substance use disorder, ^c^ n (%)	—	—	4425 (39)	207 (41)
HIV coinfection, n (%)	—	—	367 (3)	24 (5)
Prior HCV treatment experience, n (%)				
Treatment-naïve	1143 (73)	744 (72)	11,299 (100)	509 (100)
Treatment-experienced	415 (27)	291 (28)	0 (0)	0 (0)

^a^ Hispanic ethnicity was not classified as a race in the ASTRAL-1, -2, and -3 studies, but was categorized as a race in the Real-World Hepatitis Surveillance Program. ^b^ GT data were not available for all patients. ^c^ The data do not distinguish between past and current opioid or substance use. BMI, body mass index; GT, genotype; HCV, hepatitis C virus; OST, opioid substitution therapy; SOF, sofosbuvir; SVR, sustained virologic response; SVR4, SVR at 4 weeks post-treatment; SVR12, SVR at 12 weeks post-treatment; VEL, velpatasvir.

**Table 2:** Timing of Viral Relapse or Reinfection in Patients Receiving Sofosbuvir (SOF)/Velpatasvir (VEL) in the Phase 3 ASTRAL-1, -2, and -3 Studies.

Patient #	HCV GT	SVR4	SVR12	Relapsed or Reinfected	HCV RNA at Time of Relapse or Reinfection (log_10_ IU/mL)	Previous Regimen	Cirrhosis	Previous Outcome
1	3a	Yes	No	Between SVR4 and SVR12	5.2	PEG + RBV	Yes	Nonresponder
2	3a	Yes	No	Between SVR4 and SVR12	6.4	None (TN)	Yes	N/A
3	3a	Yes	No	Between SVR4 and SVR12	6.7	PEG + RBV	No	Relapse/ breakthrough
4	1a	No	No	Before SVR4	5.9	None (TN)	No	N/A
5	1b	No	No	Before SVR4	5.7	PEG + RBV	Yes	Nonresponder
6	3a	No	No	Before SVR4	6.4	None (TN)	Yes	N/A
7	3a	No	No	Before SVR4	4.9	PEG + RBV	No	Relapse/ breakthrough
8	3a	No	No	Before SVR4	6.3	None (TN)	No	N/A
9	3a	No	No	Before SVR4	5.5	PEG + RBV	Yes	Relapse/ breakthrough
10	3	No	No	Before SVR4	1.7	PEG + RBV	No	Relapse/ breakthrough
11	3a	No	No	Before SVR4	5.8	PEG + RBV	Yes	Nonresponder
12	3a	No	No	Before SVR4	2.8	None (TN)	Yes	N/A
13	3a	No	No	Before SVR4	5.1	PEG + RBV	Yes	Nonresponder

GT, genotype; HCV, hepatitis C virus; N/A, not applicable; PEG, pegylated interferon; RBV, ribavirin; SVR, sustained virologic response; SVR4, SVR at 4 weeks post-treatment; SVR12, SVR at 12 weeks post-treatment; TN, treatment-naïve.

**Table 3:** Sustained Virologic Response (SVR) Outcomes of Patients in the Phase 3 ASTRAL Studies and the Real-World Hepatitis Surveillance Program.

	Achieved SVR	Virologic Relapse or Reinfection
ASTRAL studies		
SVR4 (n = 1015)	1005 (99)	10 (1)
SVR12 (n = 1015)	1002 (99)	13 (1)
Real-World Hepatitis Surveillance Program		
*All patients with HCV treated with SOF/VEL*		
SVR4 (n = 702)	659 (94)	43 (6)
SVR12 (n = 8005)	7405 (93)	600 (7)
*Patients with both SVR4 and SVR12 data*		
SVR4 (n = 509)	479 (94)	30 (6)
SVR12 (n = 509)	485 (95)	24 (5)

Values in the table reflect n (%). HCV, hepatitis C virus; SOF, sofosbuvir; SVR4, SVR at 4 weeks post-treatment; SVR12, SVR at 12 weeks post-treatment; VEL, velpatasvir.

**Table 4:** Demographics of Patients Who Did Not Achieve Sustained Virologic Response (SVR) in the Real-World Hepatitis Surveillance Program.

	Achieved SVR4; Did Not Achieve SVR12 n = 7	Did Not Achieve SVR4; Achieved SVR12 n = 13	Did Not Achieve SVR4 or SVR12 n = 17
Age, years, mean (SD)	46 (14.6)	45 (14.3)	47 (11.5)
Sex, n (%)			
Male	4 (57)	12 (92)	15 (88)
Female	3 (43)	1 (8)	2 (12)
Race, n (%)			
Asian	0 (0)	0 (0)	0 (0)
Black	3 (43)	1 (8)	6 (35)
Hispanic	1 (14)	1 (8)	3 (18)
White	3 (43)	11 (85)	8 (47)
Other	0 (0)	0 (0)	0 (0)
Cirrhosis, n (%)	1 (14)	2 (15)	4 (24)
Opioid use disorder, ^a^ n (%)	5 (71)	6 (46)	10 (59)
Substance use disorder, ^a^ n (%)	3 (43)	4 (31)	10 (59)
HIV coinfection, n (%)	2 (29)	1 (8)	2 (12)
Prior HCV treatment experience, n (%)			
Treatment-naïve	7 (100)	13 (100)	17 (100)
Treatment-experienced	0 (0)	0 (0)	0 (0)

^a^ The data do not distinguish between past and current opioid or substance use. HCV, hepatitis C virus; SVR4, SVR at 4 weeks post-treatment; SVR12, SVR at 12 weeks post-treatment.

## Data Availability

The original contributions presented in this study are included in the article. Further inquiries can be directed to the corresponding author.

## Abbreviations

The following abbreviations are used in this manuscript:

AASLD-IDSA	American Association for the Study of Liver Diseases and the Infectious Diseases Society of America
BMI	Body mass index
DAA	Direct-acting antiviral
EASL	European Association for the Study of the Liver
GT	Genotype
HCV	Hepatitis C virus
INHSU	International Network on Health and Hepatitis in Substance Users
LTFU	Loss to follow-up
N/A	Not applicable
NPV	Negative predictive value
NS5B	Nonstructural protein 5B
OST	Opioid substitution therapy
PEG	Pegylated interferon
PPV	Positive predictive value
RBV	Ribavirin
RCT	Randomized clinical trials
SOF	Sofosbuvir
STI	Sexually transmitted infection
SVR	Sustained virologic response
SVR4	Sustained virologic response with undetectable hepatitis C virus RNA at 4 weeks after treatment completion
SVR12	Sustained virologic response with undetectable hepatitis C virus RNA at 12 weeks after treatment completion
SVR24	Sustained virologic response with undetectable hepatitis C virus RNA at 24 weeks after treatment completion
TN	Treatment-naïve
VEL	Velpatasvir
WHO	World Health Organization

## References

[1] Martinello M., Solomon S.S., Terrault N.A., Dore G.J. (2023). Hepatitis C. Lancet.

[2] World Health Organization Hepatitis C. https://www.who.int/news-room/fact-sheets/detail/hepatitis-c.

[3] Asselah T., Marcellin P., Schinazi R.F. (2018). Treatment of hepatitis C virus infection with direct-acting antiviral agents: 100% cure?. Liver Int..

[4] World Health Organization Global Health Strategy on Viral Hepatitis 2016–2021. https://iris.who.int/bitstream/handle/10665/246177/WHO-HIV-2016.06-eng.pdf?sequence=1.

[5] European Association for the Study of the Liver (2020). EASL recommendations on treatment of hepatitis C: Final update of the series. J. Hepatol..

[6] World Health Organization Updated Recommendations on Treatment of Adolescents and Children with Chronic HCV Infection, and HCV Simplified Service Delivery and Diagnostics. https://iris.who.int/bitstream/handle/10665/363590/9789240052734-eng.pdf?sequence=1.

[7] Ghany M.G., Morgan T.R., AASLD-IDSA Hepatitis C Guidance Panel (2020). Hepatitis C guidance 2019 update: American Association for the Study of Liver Diseases-Infectious Diseases Society of America recommendations for testing, managing, and treating hepatitis C virus infection. Hepatology.

[8] World Health Organization Guidelines for the Care and Treatment of Persons Diagnosed with Chronic Hepatitis C Virus Infection. https://iris.who.int/bitstream/handle/10665/273174/9789241550345-eng.pdf?ua=1.

[9] Weisberg I.S., Jacobson I.M. (2017). A pangenotypic, single tablet regimen of sofosbuvir/velpatasvir for the treatment of chronic hepatitis C infection. Expert. Opin. Pharmacother..

[10] Abraham G.M., Obley A.J., Humphrey L.L., Qaseem A., Scientific Medical Policy Committee of the American College of Physicians (2021). World Health Organization guidelines on treatment of hepatitis C virus infection: Best practice advice from the American College of Physicians. Ann. Intern. Med..

[11] Terrault N.A. (2018). Care of patients following cure of hepatitis C virus infection. Gastroenterol. Hepatol..

[12] Aleman S., Soderholm J., Busch K., Kovamees J., Duberg A.S. (2020). Frequent loss to follow-up after diagnosis of hepatitis C virus infection: A barrier towards the elimination of hepatitis C virus. Liver Int..

[13] Darvishian M., Wong S., Binka M., Yu A., Ramji A., Yoshida E.M., Wong J., Rossi C., Butt Z.A., Bartlett S. (2020). Loss to follow-up: A significant barrier in the treatment cascade with direct-acting therapies. J. Viral Hepat..

[14] Isfordink C.J., van Dijk M., Brakenhoff S.M., Kracht P.A.M., Arends J.E., de Knegt R.J., van der Valk M., Drenth J.P.H., Celine Study Group (2022). Hepatitis C Elimination in the Netherlands (CELINE): How nationwide retrieval of lost to follow-up hepatitis C patients contributes to micro-elimination. Eur. J. Intern. Med..

[15] Lazarus J.V., Villota-Rivas M., Fernandez I., Gea F., Ryan P., Lopez S.A., Guy D., Calleja J.L., Garcia-Samaniego J. (2022). A cascade of care analysis on the elimination of hepatitis C from public hospitals in Madrid. Commun. Med..

[16] Mendes L.C., Ralla S.M., Vigani A.G. (2016). Loss to follow-up in anti-HCV-positive patients in a Brazilian regional outpatient clinic. Braz. J. Med. Biol. Res..

[17] Zarebska-Michaluk D., Brzdek M., Tronina O., Janocha-Litwin J., Sitko M., Piekarska A., Klapaczynski J., Parfieniuk-Kowerda A., Sobala-Szczygiel B., Tudrujek-Zdunek M. (2024). Loss to follow-up of patients after antiviral treatment as an additional barrier to HCV elimination. BMC Med..

[18] Ziff J., Vu T., Dvir D., Riazi F., Toribio W., Oster S., Sigel K., Weiss J. (2021). Predictors of hepatitis C treatment outcomes in a harm reduction-focused primary care program in New York City. Harm Reduct. J..

[19] Alenzi M., Almeqdadi M. (2024). Bridging the gap: Addressing disparities in hepatitis C screening, access to care, and treatment outcomes. World J. Hepatol..

[20] Degenhardt L., Peacock A., Colledge S., Leung J., Grebely J., Vickerman P., Stone J., Cunningham E.B., Trickey A., Dumchev K. (2017). Global prevalence of injecting drug use and sociodemographic characteristics and prevalence of HIV, HBV, and HCV in people who inject drugs: A multistage systematic review. Lancet Glob. Health.

[21] Facente S.N., Grinstein R., Bruhn R., Kaidarova Z., Wilson E., Hecht J., Burk K., Grebe E., Morris M.D. (2022). Hepatitis C prevalence and key population size estimate updates in San Francisco: 2015 to 2019. PLoS ONE.

[22] Valerio H., Alavi M., Silk D., Treloar C., Martinello M., Milat A., Dunlop A., Holden J., Henderson C., Amin J. (2021). Progress towards elimination of hepatitis C infection among people who inject drugs in Australia: The ETHOS Engage study. Clin. Infect. Dis..

[23] Singh G.P., Lata S., Swu A.K., Virk N.S., Singh J., Thakkar S. (2022). A retrospective study to find the prevalence of HIV, HCV, and dual HIV-HCV infection in the prison inmates. J. Family Med. Prim. Care.

[24] Dolan K., Wirtz A.L., Moazen B., Ndeffo-Mbah M., Galvani A., Kinner S.A., Courtney R., McKee M., Amon J.J., Maher L. (2016). Global burden of HIV, viral hepatitis, and tuberculosis in prisoners and detainees. Lancet.

[25] Burgess S.V., Hussaini T., Yoshida E.M. (2016). Concordance of sustained virologic response at weeks 4, 12 and 24 post-treatment of hepatitis c in the era of new oral direct-acting antivirals: A concise review. Ann. Hepatol..

[26] Martinot-Peignoux M., Stern C., Maylin S., Ripault M.P., Boyer N., Leclere L., Castelnau C., Giuily N., El Ray A., Cardoso A.C. (2010). Twelve weeks posttreatment follow-up is as relevant as 24 weeks to determine the sustained virologic response in patients with hepatitis C virus receiving pegylated interferon and ribavirin. Hepatology.

[27] Chen J., Florian J., Carter W., Fleischer R.D., Hammerstrom T.S., Jadhav P.R., Zeng W., Murray J., Birnkrant D. (2013). Earlier sustained virologic response end points for regulatory approval and dose selection of hepatitis C therapies. Gastroenterology.

[28] Food and Drug Administration Center for Drug Evaluation and Research (CDER) Chronic Hepatitis C Virus Infection: Developing Direct-Acting Antiviral Drugs for TREATMENT guidance for Industry. https://www.fda.gov/files/drugs/published/Chronic-Hepatitis-C-Virus-Infection---Developing-Direct-Acting-Antiviral-Drugs-for-Treatment-Guidance-for-Industry.pdf.

[29] Gane E., de Ledinghen V., Dylla D.E., Rizzardini G., Shiffman M.L., Barclay S.T., Calleja J.L., Xue Z., Burroughs M., Gutierrez J.A. (2021). Positive predictive value of sustained virologic response 4 weeks posttreatment for achieving sustained virologic response 12 weeks posttreatment in patients receiving glecaprevir/pibrentasvir in phase 2 and 3 clinical trials. J. Viral Hepat..

[30] Lin C.P., Liang P.C., Huang C.I., Yeh M.L., Hsu P.Y., Hsu C.T., Wei Y.J., Liu T.W., Hsieh M.Y., Hou N.J. (2021). Concordance of SVR12, SVR24, and SVR durability in Taiwanese chronic hepatitis C patients with direct-acting antivirals. PLoS ONE.

[31] Liu C.H., Chang Y.P., Lee J.Y., Chen C.Y., Kao W.Y., Lin C.L., Yang S.S., Shih Y.L., Peng C.Y., Lee F.J. (2024). Four weeks of off-treatment follow-up is sufficient to determine virologic responses at off-treatment week 12 in patients with hepatitis C virus infection receiving fixed-dose pangenotypic direct-acting antivirals. J. Med. Virol..

[32] Milosevic I., Filipovic A., Beronja B., Mitrovic N., Ruzic M., Simic J., Knezevic N., Pete M., Todorovic N., Nikolic N. (2024). Optimizing hepatitis C treatment monitoring: Is sustained virologic response at 4 weeks becoming the new standard?. Microorganisms.

[33] Hepatitis C Virus Infection Consensus Statement Working Group. Australian Recommendations for the Management of Hepatitis C Virus Infection: A Consensus Statement. https://www.hepcguidelines.org.au/wp-content/uploads/2022/11/hepatitis-C-virus-infection-a-consensus-statement-2022.pdf.

[34] Alghamdi A.S., Alghamdi H., Alserehi H.A., Babatin M.A., Alswat K.A., Alghamdi M., AlQutub A., Abaalkhail F., Altraif I., Alfaleh F.Z. (2024). SASLT guidelines: Update in treatment of hepatitis C virus infection, 2024. Saudi J. Gastroenterol..

[35] Sheehan Y., Akhil G., Sheehan J., Maduka N., Altice F.L., Alves da Costa F., Cox S., Elsharkawy A.M., Salah E., Stoové M. Global Guidelines for Viral Hepatitis Service Delivery in Prisons. https://inhsu.org/wp-content/uploads/2024/09/Global-Guidelines_FINAL.pdf.

[36] Aronsohn A. AASLD/IDSA guidance update: New SVR guidance and test and treat algorithm. Proceedings of the AASLD: The Liver Meeting.

[37] American Association for the Study of Liver Diseases and the Infectious Diseases Society of America Hepatitis C Test and Treat Follow up Visit. https://www.hcvguidelines.org/sites/default/files/full-guidance-pdf/HCV%20Test%20and%20Treat%20Final%20011725.pdf.

[38] Razavi H.A., Waked I., Qureshi H., Kondili L.A., Duberg A.S., Aleman S., Tanaka J., Lazarus J.V., Low-Beer D., Abbas Z. (2025). Number of people treated for hepatitis C virus infection in 2014-2023 and applicable lessons for new HBV and HDV therapies. J. Hepatol..

[39] Feld J.J., Jacobson I.M., Hezode C., Asselah T., Ruane P.J., Gruener N., Abergel A., Mangia A., Lai C.L., Chan H.L. (2015). Sofosbuvir and velpatasvir for HCV genotype 1, 2, 4, 5, and 6 infection. N. Engl. J. Med..

[40] Foster G.R., Afdhal N., Roberts S.K., Brau N., Gane E.J., Pianko S., Lawitz E., Thompson A., Shiffman M.L., Cooper C. (2015). Sofosbuvir and velpatasvir for HCV genotype 2 and 3 infection. N. Engl. J. Med..

[41] Curry M.P., O’Leary J.G., Bzowej N., Muir A.J., Korenblat K.M., Fenkel J.M., Reddy K.R., Lawitz E., Flamm S.L., Schiano T. (2015). Sofosbuvir and velpatasvir for HCV in patients with decompensated cirrhosis. N. Engl. J. Med..

[42] Wyles D., Brau N., Kottilil S., Daar E.S., Ruane P., Workowski K., Luetkemeyer A., Adeyemi O., Kim A.Y., Doehle B. (2017). Sofosbuvir and velpatasvir for the treatment of hepatitis C virus in patients coinfected with human immunodeficiency virus type 1: An open-label, phase 3 study. Clin. Infect. Dis..

[43] Buggisch P., Wursthorn K., Stoehr A., Atanasov P.K., Supiot R., Lee J., Ting J., Petersen J. (2019). Real-world effectiveness and safety of sofosbuvir/velpatasvir and ledipasvir/sofosbuvir hepatitis C treatment in a single centre in Germany. PLoS ONE.

[44] Charatcharoenwitthaya P., Wongpaitoon V., Komolmit P., Sukeepaisarnjaroen W., Tangkijvanich P., Piratvisuth T., Sanpajit T., Sutthivana C., Bunchorntavakul C., Sobhonslidsuk A. (2020). Real-world effectiveness and safety of sofosbuvir and nonstructural protein 5A inhibitors for chronic hepatitis C genotype 1, 2, 3, 4, or 6: A multicentre cohort study. BMC Gastroenterol..

[45] Huang Y.T., Hsieh Y.Y., Chen W.M., Tung S.Y., Wei K.L., Shen C.H., Chang K.C., Lu C.K., Yen C.W., Lu S.N. (2021). Sofosbuvir/velpatasvir is an effective treatment for patients with hepatitis C and advanced fibrosis or cirrhosis in a real-world setting in Taiwan. BMC Gastroenterol..

[46] Liu C.H., Chen P.Y., Chen J.J., Lo C.C., Su W.W., Tseng K.C., Liu C.J., Huang C.S., Huang K.J., Yang S.S. (2021). Sofosbuvir/velpatasvir for patients with chronic hepatitis C virus infection and compensated liver disease: Real-world data in Taiwan. Hepatol. Int..

[47] Mangia A., Milligan S., Khalili M., Fagiuoli S., Shafran S.D., Carrat F., Ouzan D., Papatheodoridis G., Ramji A., Borgia S.M. (2020). Global real-world evidence of sofosbuvir/velpatasvir as simple, effective HCV treatment: Analysis of 5552 patients from 12 cohorts. Liver Int.

[48] Wilton J., Wong S., Yu A., Ramji A., Cook D., Butt Z.A., Alvarez M., Binka M., Darvishian M., Jeong D. (2020). Real-world effectiveness of sofosbuvir/velpatasvir for treatment of chronic hepatitis C in British Columbia, Canada: A population-based cohort study. Open Forum Infect. Dis..

[49] Younossi Z.M., Stepanova M., Feld J., Zeuzem S., Jacobson I., Agarwal K., Hezode C., Nader F., Henry L., Hunt S. (2016). Sofosbuvir/velpatasvir improves patient-reported outcomes in HCV patients: Results from ASTRAL-1 placebo-controlled trial. J. Hepatol..

[50] Younossi Z.M., Stepanova M., Gordon S., Zeuzem S., Mann M.P., Jacobson I., Bourliere M., Cooper C., Flamm S., Reddy K.R. (2018). Patient-reported outcomes following treatment of chronic hepatitis C virus infection with sofosbuvir and velpatasvir, with or without voxilaprevir. Clin. Gastroenterol. Hepatol..

[51] Sarrazin C., Isakov V., Svarovskaia E.S., Hedskog C., Martin R., Chodavarapu K., Brainard D.M., Miller M.D., Mo H., Molina J.M. (2017). Late relapse versus hepatitis C virus reinfection in patients with sustained virologic response after sofosbuvir-based therapies. Clin. Infect. Dis..

[52] Han W.M., Solomon S., Smeaton L., Avihingsanon A., Wagner-Cardoso S., Li J., Parvangada A., Sulkowski M., Naggie S., Martin R. (2024). Reinfection and resistance associated substitutions following a minimal monitoring approach for HCV treatment in MINMON trial. Clin. Infect. Dis..

[53] Litwin A.H., Tsui J.I., Heo M., Mehta S.H., Taylor L.E., Lum P.J., Feinberg J., Kim A.Y., Norton B.L., Pericot-Valverde I. (2024). Hepatitis C virus reinfection among people who inject drugs: Long-term follow-up of the HERO study. JAMA Netw. Open.

[54] Simmons B., Saleem J., Hill A., Riley R.D., Cooke G.S. (2016). Risk of late relapse or reinfection with hepatitis C virus after achieving a sustained virological response: A systematic review and meta-analysis. Clin. Infect. Dis..

[55] Kuriry H., Casey J., Krassenburg L., La D., Kuczynski M., Shah H., Janssen H.L.A., Hansen B.E., Feld J.J. (2021). Spontaneous clearance after relapse following direct-acting antiviral treatment for chronic HCV infection. Clin. Gastroenterol. Hepatol..

